# Is there any correlation between Estradiol supplementation, as luteal phase support, and clinical pregnancy in ART cycles? A cross-sectional study

**DOI:** 10.18502/ijrm.v13i11.7964

**Published:** 2020-11-22

**Authors:** Maryam Eftekhar, Banafsheh Mohammadi, Esmat Mangoli, Maryam Mortazavi

**Affiliations:** ^1^Department of Obstetrics and Gynecology, Research and Clinical Center for Infertility, Shahid Sadoughi University of Medical Sciences, Yazd, Iran.; ^2^Department of Reproductive Biology, Research and Clinical Center for Infertility, Shahid Sadoughi University of Medical Sciences, Yazd, Iran.; ^3^Department of Gynecology and Obstetrics, Rafsanjan University of Medical Sciences, Rafsanjan, Iran.

**Keywords:** Luteal phase, Estradiol, Progesterone, ICSI, Pregnancy rate.

## Abstract

**Background:**

Endometrial receptivity is one of the important factors in assisted reproductive technology (ART) success. In the luteal phase of an ART cycle, serum estradiol (E2) and progesterone are often placed in low levels. Supporting the luteal phase with progesterone is a usual method.

**Objective:**

To evaluate the effects of E2 supplementation plus progesterone on the luteal phase support in the antagonist protocol who have undergone intracytoplasmic sperm injection-embryo transfer cycles.

**Materials and Methods:**

In this cross-sectional study, 200 patients with antagonist stimulation protocol, who had undergone intracytoplasmic sperm injection treatment, were divided into two groups based on the use of E2 supplementation. In both groups, 400 mg progesterone suppositories (CyclogestⓇ), twice a day/vaginally, was administered starting from the day of oocyte collection until the fetal heart activity. However, in the E2 group, in addition to progesterone, 4 mg tablet of E2 was received daily. Beta hCG was checked 14 days after the embryo transfer, and the clinical pregnancy rate was the main endpoint.

**Results:**

The patients' characteristics were matched, and insignificant differences were observed, except for endometrial thickness. The clinical outcomes showed the rate of pregnancy was higher in the E2 group compared to the control group; nonetheless, statistically, there was no noticeable difference.

**Conclusion:**

E2 supplementation had no beneficial effect in the luteal phase support of IVF cycles. Nevertheless, more studies are required to confirm the supportive role of E2 supplementation for embryo implantation and to improve the outcomes in ART cycles.

## 1. Introduction

Assisted Reproductive Technology (ART) has been used worldwide for many years and embryo implantation is a main factor of this program. Endometrial receptivity is one of the important attributes in the success of ART (1). During controlled ovarian hyperstimulation, supraphysiologic levels of estradiol (E2) causes a decrease in the luteinizing hormone levels, following which the corpus luteum may be inactivated in the absence of luteinizing hormone (2). Unfortunately, the use of Gonadotropin-releasing Hormone (GnRH) analogs also causes the inhibition of corpus luteum in these cycles. In addition, during oocyte retrieval, the granulosa cells curettage, as a consequence of which both corpus luteum function and progesterone production decrease. In the luteal phase of an ART cycle, serum E2 and progesterone often fall too low levels (3). The incidence and maintenance of pregnancy require suitable secretion of P from the corpus luteum (4). Therefore, a progesterone supplement is run during the luteal phase to reach the ideal endometrial receptivity. There are various protocols of luteal support in the ART cycles. particularly, the luteal phase support (LPS) with progesterone, as a usual method (2). E2 plays an important role in the proliferation of the epithelial, stromal, and uterine vessels in the follicular phase of the menstrual cycle (5). Beckers and coworkers showed that a mid-luteal decrease of estrogen occurs following the luteal vaginal bleeding, which may be related to implantation failure (6). The benefit of additional LPS with E2 is not clear; some studies have found out that the a co-administration of E2 with P in the luteal phase increases the implantation and pregnancy rates in the women who have undergone in vitro fertilization/intracytoplasmic sperm injection-embryo transfer (IVF/ICSI-ET) cycles, as compared to progesterone alone (2).

The key objective of the present study was to evaluate the effect of E2 supplementation plus to progesterone for the LPS in antagonist protocol on the clinical outcomes of patients who have undergone ICSI-ET cycles.

## 2. Materials and Methods

### Patients

In this cross-sectional study, 200 women who referred to Yazd Research and Clinical Center for Infertility during one year from April 2018 to April 2019 were treated with ICSI-ET with controlled ovarian stimulation by antagonist protocol were selected. Among them, only those who were in the Fresh embryo transfer cycle were studied. According to the LPS protocol, patients were divided into two groups as a control group that received progesterone alone and the E2 group which received E2+progesterone.

Women with the risk of ovarian hyperstimulation syndrome, history of endometriosis, surgical hysteroscopy, having more than two implantation procedures, and severe male factor were excluded.

### Treatment protocol

The stimulation protocol was started from the second day of the cycle with injecting 150-225 IU recombinant human follicle-stimulating hormone (Gonal-F Serono or Pergoveris, Merck-Serono, Germany) for five days. On the 6${}^{\mathrm{th}}$ day, follicles size and medication response were evaluated through transvaginal sonography. After confirming the mature follicle (≥ 14 mm) by transvaginal sonography, 0.25 mg/daily GnRH antagonist (Cetrotide, Merck-Serono, Germany) was injected subcutaneously. hCG (5000-10000 IU) (Pregnyl, Organon, Netherland) was injected when at least two follicles with a mean diameter of 17 mm were observed. Oocyte pick up was done under sedation36 hr after triggering. ICSI was done for all mature oocytes.

Embryo transfers were performed with a soft COOK Medical embryo transfer catheter (COOK, USA) and ultrasound guidance on the second day. Two good-quality embryos (grade A, and B) were transferred. In both groups, 400 mg of progesterone suppositories (CyclogestⓇ, Barnstaple, UK) was started twice a day from the day of oocyte retrieval and continued until the detection of fetal heart activity. However, in the E2 group, 2 mg oral tablet of E2 valerate (Aburaihan Co., Tehran, Iran), was given twice a day from the day of embryo transfer and continued until the fetal heart activity.

Chemical pregnancy was assessed by measuring the serum beta-hCG (β-hCG) on day 14 after the embryo transfer. A positive pregnancy test was definitive when β-hCG > 50 IU/L. Clinical pregnancies were confirmed when fetal heart activity was determined by transvaginal ultrasonography 2-3 wk after positive β-hCG. The implantation rate was defined as the gestational sacs to the number of the embryos transferred ratio.

### Ethical consideration

The study protocol was approved by the ethics committee of the Research and Clinical Center for Infertility, Yazd, Iran (Code: IR.SSU.RSI.REC.1398.028). Written consent forms were signed by all participants prior to the study.

### Statistical analysis 

Data were presented as Mean ± SD and analyzed using the SPSS version 20 (Statistical Package for the Social Sciences, SPSS, Chicago, IL). The normality of data was checked with the Shapiro test. Independent *t* test and Pearson's Chi-square test (χ2) were used to compare the groups. P < 0.05 was considered as statistically significant.

## 3. Results

Results showed that there was no statistically significant difference between groups except for the endometrial thickness (p = 0.048) (Table I). For each group, the patients were selected from different etiologies and there was no difference between them (Table II). In comparison of clinical outcomes the results showed that the rate of pregnancy was higher in E2 groups; however, statistically, it was insignificant (Table III).

**Table 1:** Comparison of patients' characteristics between the two groups


**Patients characteristics**	**Estradiol group **	**Control group **	**P-value**
**Age**	31.35 ± 4.66	31.03 ± 4.6	0.627
**BMI**	25.46 ± 2.93	23.36 ± 1.33	0.318
**Duration infertility **	5.9 ± 3.57	6.02 ± 3.21	0.797
**AMH**	2.29 ± 1.06	2.66 ± 1.5	0.196
**Embryo **	2.62 ± 1.31	2.87 ± 1.41	0.198
**Num. embryo transfer**	1.82 ± 0.38	1.79 ± 0.40	0.595
**COC**	6.72 ± 2.82	6.23 ± 2.39	0.187
**MII**	4.19 ± 1.96	4.05 ± 1.84	0.604
**Estradiol**	1017.8 ± 345.8	1054.5 ± 404.9	0.492
**Endometrial thickness**	9.05 ± 1.18	9.44 ± 1.34	0.048${}^{*}$
**Duration of stimulation **	13 ± 1.93	13. 1 ± 2.00	0.051
**Gonadotropin dose**	2834071.5 ± 892655.34	2921067.0 ± 681763.18	0.440
Data presented as Mean ± SD. Independent* t* test and; *p-value < 0.05 was significant AMH: Anti-mullerian hormone; COC: Cumulus oocyte complex; MII: Metaphase II; BMI: Body mass index			

**Table 2:** Comparison of different etiologies between groups


**Variables **	**MF**	**PCO**	**OF**	**TF**	**Unknown**	**Mixed**	**P-value**
**Estradiol group**	29 (29)	9 (9)	21 (21)	4 (4)	20 (20)	17 (17)	
**Control group**	28 (28)	13 (13)	24 (24)	1 (1)	18 (18)	16 (16)	<brow>-2</erow> 0.718
Data presented as percentage. Chi-square test, MF: Male factor; PCO: Polycystic ovary; OF: Ovarian factor; TF: Tubal factor							

**Table 3:** Comparison of pregnancy outcomes between the two groups


**Variables**	**Estradiol group (n = 100)**	**Control group (n = 100)**	**OR (CI)**	**P-value**
**Chemical pregnancy**	33 (33)	28 (28)	1.26 (0.69-2.31)	0.539
**Clinical pregnancy**	26 (26)	21 (21)	1.32 (0.68-2.54)	0.505
Data presented as percentage. Chi-square test, OR: Odds ratio; CI: Confidence interval				

## 4. Discussion

The result of this study showed that endometrial thickness was statistically different between the groups that according to our previous study this difference was negligible (7). In the present study, we aimed to compare the efficacy of adding E2 to progesterone in LPS in antagonist protocols and evaluate ICSI outcomes. The results showed that a co-administration of progesterone and E2 in the luteal phase had a similar result as with only progesterone supplementation. While several studies have evaluated the effect of LPS of estrogen supplementation on the pregnancy rate, it is still a debated matter (3, 5, 8). Some studies assessed the effect of estrogen supplementation on agonist IVF cycles (9). However, in antagonist IVF cycles, it is still a debate. Drakakis and coworkers evaluated the effect of E2 in the luteal phase in ICSI patients and reported that E2 not only had a positive effect on the pregnancy outcome but also has no adverse effects (10). Also, Zhang and colleague in a systematic review and meta-analysis showed a higher clinical pregnancy rate (CPR) in progesterone plus estrogen for LPS compare to progesterone alone in IVF cycles (11). In contrast, Huang and colleagues in a meta-analysis stated that oral estrogens supplementation for luteal phase in agonist IVF cycles did not improve the IVF/ICSI outcomes (3).

Some studies concluded that there was no benefit of adding E2 similar to our study (8, 12-15). Çakar and coworker evaluated patients in the ICSI cycles and the antagonist protocol. Progesterone 90 mg vaginal gel once a day and micronized E2 4 mg/day were started from the day of oocyte collection and continued till the 12${}^{\mathrm{th}}$ day of embryo transfer. They revealed that the addition of E2 to LPS had no valuable outcome on CPR in antagonist IVF cycles (8). Munjal and coworker, in a group of women who had undergone the controlled ovarian stimulation by gonadotropin and GnRH antagonist protocol, determined that giving E2 supplementation along with progesterone in the luteal phase did not improve the pregnancy rates significantly (13). Ismail Madkour and colleagues also reported similar outcome and concluded that the daily addition of 4 mg estrogen for luteal support in ART cycle, using the antagonist protocol, did not increase the pregnancy outcomes (16). The effect of adding E2 in the luteal phase may be dose-dependent. Lukaszuk and colleagues confirmed a meaningfully higher CPR in a group accompanied with 6 mg E2 compared to a group complemented with 2 mg E2 and the group without E2 (17).

A few previous studies have considered whether or not E2 supplementation is useful in patients with different ovarian responses and serum E2 levels (3, 10). Zhao and co-workers in a retrospective cohort study concluded that the effects of adding E2 for luteal phase depended on the E2 levels on the hCG trigger day. E2 supplementation was linked to improved outcomes in patients with low E2 levels; however, it was harmful in persons with high E2 levels on the trigger day. They presented that E2 supplementation in cases with E2 < 5,000 pmol/L on the day of hCG trigger considerably increased their live birth rate (4). Kasapoglu and colleague assessed the clinical outcomes of patients who had a ratio of serum E2 levels to the oocytes number < 100 pg/ml and received E2 supplementation during the luteal phase. They concluded that implantation and CPR per embryo transfer following the transfer of a single embryo did not increase after addition of E2 in the luteal phase (18). On the other hand, Kutlusoy and colleagues evaluated this program (E2 + progestin) in IVF cycles with poor responder patients. They showed that adding 2 mg/day E2 in addition to P for luteal support meaningfully increased CPRs in such patients (19).

## 5. Conclusion

In conclusion, the supportive role of E2 in improving embryo implantation and pregnancy is unclear and needs further studies. Although the results of this study exhibited no useful effect of E2 supplementation in the luteal phase of IVF cycles, estrogen supplementation may be useful in some subgroups of the patients. Therefore, a large RCT study is needed to further clear up the role of luteal E2 supplementation in IVF cycles, as well as the optimal regimen (dose and route), not well defined to the date.

##  Conflict of Interest 

The authors have no conflict of interest to declare.
